# Transforming perinatal health with AI-predictive models in precision and digital innovations for maternal and fetal well-being: a systematic review

**DOI:** 10.1093/oodh/oqag020

**Published:** 2026-08-04

**Authors:** Shahrzad Kaveh, Zahra Khedri, Solmaz Sohrabei

**Affiliations:** Department of Midwifery, School of Nursing and Midwifery, Ayatollah Mousavi Hospital, Zanjan University of Medical Sciences, Sabooti Street 4513956184, Zanjan, Iran; Department of Obstetrics and Gynecology, Ziaeian Hospital, Tehran University of Medical Sciences, Qazvin Street 1366736511, Tehran, Iran; Student Research Committee (Department and Faculty of Health Information Technology and Management, Medical Informatics, School of Allied Medical Sciences), Shahid Beheshti University of Medical Sciences, Darband Street 197653213, Tehran, Iran

**Keywords:** transforming perinatal health, machine learning, deep learning, mobile health, maternal and fetal well-being

## Abstract

The convergence of artificial intelligence (AI) and wearable monitoring devices is revolutionizing the management of high-risk pregnancies, offering unprecedented opportunities for early detection, personalized care, and improved outcomes. These technologies address critical challenges in maternal and fetal health, especially in resource-limited settings, by enabling proactive and continuous care. This systematic review specifically focuses on the integration of artificial intelligence and wearable devices for managing high-risk pregnancies and highlights regulatory and ethical challenges. Databases like PubMed (Medline), Scopus, and Web of Science were explored for pertinent studies (from 2015 to 2024). Articles were chosen based on specific inclusion and exclusion criteria, and essential data including technology type, AI algorithms employed, and their influence on perinatal care were collected. The Risk of Bias in the studies was evaluated utilizing standard assessment tools such as PROBAST+AI, and study quality was assessed with TRIPOD+AI. Ultimately, the findings were examined through narrative analysis, leading to the identification of challenges and future research directions. This systematic review examined 16 studies focusing on the application of AI and digital innovations in maternal and fetal healthcare. The studies investigated various AI techniques, including machine-learning models for predicting risks to maternal health, deep learning for monitoring fetal well-being, and wearable sensor technologies for remote health tracking. Many studies produced encouraging outcomes, with AI models reaching high levels of accuracy in early detection, risk assessment, and real-time patient monitoring. However, issues such as dataset biases, generalizability, and ethical concerns continue to pose significant challenges. Additional research is necessary to validate these AI-driven methods across diverse populations to ensure equitable and effective healthcare outcomes. The integration of technologies has the potential to significantly improve the management of high-risk pregnancies. However, a critical regulatory gap exists that must be addressed to ensure that these technologies are used ethically, safely, and effectively. This will involve the development of new regulatory frameworks, a focus on transparency and accountability, and a commitment to addressing bias and data security to provide the best care possible. AI has the potential to transform maternity services by improving risk prediction, enabling remote monitoring, and supporting clinical decisions. However, realizing this potential requires addressing limitations related to data quality, algorithm accuracy, user privacy, and ethical considerations.

## Introduction

A high-risk pregnancy (HRP) is generally defined as one where there is an increased chance of complications for the mother, the fetus, or both. These complications can lead to a range of adverse outcomes, including maternal morbidity and mortality, fetal growth restriction, preterm birth, and stillbirth [1]. Identifying and monitoring HRPs is crucial for enabling timely interventions and improving outcomes for both the mother and the child. HRPs include complications. Such as fetal growth restriction, preterm birth, gestational diabetes, preeclampsia (PE), and risks associated with pre-existing maternal conditions, as well as other factors like maternal age and lifestyle. Several factors can contribute to a pregnancy being classified as high-risk. Worldwide, around 20 million pregnancies are deemed high-risk, resulting in nearly 800 maternal fatalities due to complications related to pregnancy [2]. In India, it is estimated that 20%–30% of pregnancies are categorized this way, leading to 70% of maternal mortality and morbidity [3].

Global initiatives aimed at improving maternal health focus largely on antenatal, intrapartum, and postnatal care to reduce the impact of maternal mortality and morbidity [4]. Data worldwide indicate that ~87% of women access healthcare services at least once during their pregnancy [5]. Antenatal care (ANC) appointments are crucial for promoting a healthy pregnancy through consistent health evaluations, specialized care during pregnancy, and identifying HRPs. The World Health Organization advises a minimum of four to eight ANC visits, while in India, at least four ANC visits are required by law [3]. Timely detection of HRPs can greatly decrease complications and diminish mortality and morbidity rates. Therefore, all expectant mothers must be screened for high risk, and those identified as high risk should receive regular follow-up care. HRPs pose significant challenges to maternal and fetal health, necessitating advanced and proactive monitoring strategies. Traditional methods of prenatal care often fall short in providing the continuous, detailed oversight needed to manage these complex cases effectively [6].

This is where the integration of wearable devices, artificial intelligence (AI), and real-time systems becomes crucial. These technologies offer the potential to transform prenatal care, particularly for those facing HRPs, by enabling continuous, personalized, and proactive monitoring. Wearable devices can provide a constant stream of physiological data from the mother, tracking vital signs, activity levels, and other relevant metrics [7]. This continuous data collection offers a more comprehensive view of a mother’s health than periodic checkups. AI can analyze these data to identify patterns, predict potential complications, and provide early warnings to healthcare providers. AI’s ability to process large datasets and detect subtle anomalies is invaluable for early identification of risk factors [8]. AI can also assist with the interpretation of medical images such as ultrasounds. Real-time systems are essential to ensure that the data collected and the insights generated are available immediately to both the expectant mother and her healthcare team. This allows for timely interventions and personalized adjustments to care plans [9]. The sources discuss the importance of user interfaces in communicating AI interpretations to healthcare practitioners in a way that is clear, accurate, and does not interrupt clinical workflow. By combining these elements, healthcare systems can provide a more responsive and effective approach to managing HRPs [10].

This integration not only enhances the quality of care but also empowers healthcare providers with the tools necessary to make informed decisions promptly; potentially leading to improved outcomes for both mothers and their babies. The aim is to move from reactive to preventative care and help to mitigate some of the dangers of HRP [11]. Khan et al. [12] conducted a systematic review that explored the use of AI in screening for adverse perinatal outcomes such as preterm birth and PE. While their work comprehensively examined machine learning (ML) and deep learning algorithms, it did not specifically address the integration of wearable devices [13]. Similarly, Armanious et al. [14] reviewed the application of AI in identifying perinatal health predictors, focusing on outcomes like preterm delivery and low birth weight; however, they did not highlight the role of wearable technologies [12]. Altuhaifa et al. [15] provided an updated systematic review on the role of AI and ML in obstetrics and midwifery, offering insights into a wide array of AI applications across maternal care. Nevertheless, their study did not emphasize the integration of AI with wearable technologies in the context of HRPs [14]. Shah et al. [16] focused on wearable sensors for maternal health monitoring, detailing device types, and their applications in tracking maternal physiological parameters. Yet, the study did not explore their combination with AI-based analytics or their specific application in HRPs [15]. In a similar vein, Zompas et al. [17] reviewed the intersection of wearable sensors, data processing, and AI in pregnancy monitoring, concentrating on technological advancements. However, their discussion of regulatory and ethical challenges and the focus on HRP contexts remained limited [16].

Taken together, although these previous studies offer valuable insights into either AI applications or wearable technology in maternal health, the specific and combined exploration of both domains AI and wearables in managing HRPs, along with considerations of regulatory and ethical issues, sets the current review apart. This systematic study aims to investigate the positive impacts of AI and wearable devices in the management of HRPs, with the goal of presenting findings and addressing a gap in the existing literature. The research will explore how AI can contribute to early risk detection, personalized care, and real-time monitoring through analysis of physiological data and medical images, such as ultrasounds and vital maternal signs. Additionally, the study will examine how wearable devices enable continuous data collection and remote monitoring of pregnant mothers. Given the absence of a systematic review on this topic, this study is designed to provide evidence-based insights into how these technologies can enhance maternal and fetal health outcomes in HRPs, moving from reactive to preventative care and addressing a gap in the literature. We developed the following three research questions:

Q1: What are the effects of AI predictive models on prenatal health?

Q2: What are AI predictive models used for in prenatal health data analysis?

Q3: What are the performance criteria for AI predictive models?

## Methods

This systematic review aims to synthesize the existing evidence on the use of AI and digital innovations in improving perinatal health outcomes for both mothers and fetuses. Given the potential of these technologies to revolutionize prenatal care, labor and delivery agreement, and postpartum care, a thorough evaluation of their effectiveness, safety, and implementation challenges is warranted. This review aims to identify best practices, assess the impact of AI on maternal and fetal health, and highlight areas for future research.

### Objectives

- To systematically identify and synthesize studies evaluating the use of AI and digital innovations in perinatal health.
- To assess the effectiveness of AI-driven interventions in improving Maternal and fetal health outcomes.
- To evaluate the safety and potential risks associated with the implementation of these technologies.
- To identify barriers and facilitators to the adoption of AI and digital Innovations in perinatal care.

### Search strategy

#### Databases

A comprehensive search was conducted in the following electronic databases (2015–24): PubMed (MEDLINE), Embase, Web of Science, Scopus (Table 1), the search strategy combined terms related to perinatal health, AI, and digital innovations.

**Table 1:** Search keywords and database summary.

Search keywords	Databases	Total in Each database	Selection based on title	Selection based on abstract	Selection based on full-text reading and quality appraisal
**(“Pregnancy” OR “Prenatal” OR “Perinatal” OR “Maternal” OR “Fetal” OR “Infant” OR “Postpartum” OR “Labor” OR “Delivery”) AND (“Artificial Intelligence” OR “AI” OR “Machine Learning” OR “Deep Learning” OR “Neural Network” OR “Algorithm” OR “Predictive Model”) AND (“Digital Health” OR “Telemedicine” OR “Wearable Devices” OR “Mobile Health” OR “mHealth” OR “eHealth” OR “Remote Monitoring” OR “Sensor” OR “App” OR “Software” OR “Platform”).**	**PubMed (including MEDLINE)**	77	33	18	4
	**Scopus**	109	39	9	5
	**Embase**	53	16	4	4
	**Web of Science**	60	17	7	3
	**Total**	299	105	38	16

### Inclusion and exclusion criteria

#### Inclusion criteria

- Studies evaluating the use of AI and digital innovations in any aspect of perinatal health (prenatal, intrapartum, or postpartum) were included.
- Study designs included randomized controlled trials, quasi-experimental studies, and cohort, case-control, cross-sectional, qualitative, and mixed methods studies.
- Participants included pregnant women, fetuses, infants, and perinatal healthcare providers.

##### Outcomes

Any outcome related to maternal or fetal health, including but not limited to:

Maternal mortality and morbidity

- Fetal growth and development
- Preterm birth
- PE
- Gestational diabetes
- Postpartum depression
- Patient satisfaction
- Healthcare utilization

#### Exclusion criteria

We excluded studies that did not involve AI or digital innovations, were solely focused on basic science (*in vitro* studies), were case reports, editorials, opinion pieces, or letters without original data, or had no full text available after reasonable contact attempts.

### Study selection

Two independent reviewers (S.S., Sh.K.) screened titles and abstracts of all identified records based on the inclusion and exclusion criteria. Full texts of potentially relevant articles were retrieved and assessed independently by two reviewers (Z.Kh. and Sh.K.). Discrepancies between reviewers were resolved through discussion and, if necessary, consultation with a third reviewer (S.S.). A PRISMA flow diagram was used to document the study selection process.

### Data extraction

A standardized data extraction form was developed and piloted on a subset of included studies. Data were extracted by two independent reviewers. Discrepancies were resolved through discussion or consultation with a third reviewer.

Extracted data included:

- Study characteristics (author, year, country, study design, sample size)
- Participant characteristics (age, parity, risk factors)
- Intervention details (type of AI/digital innovation, description of the intervention, implementation setting)
- Control/comparison group details

### Quality assessment and risk of bias

In this systematic review, 16 articles were chosen for evaluation. The risk of bias in the included studies was assessed using established evaluation frameworks, including PROBAST+AI, while study quality was examined based on TRIPOD+AI guidelines. The Prediction Model Risk of Bias Assessment Tool (PROBAST) is designed to evaluate the quality, potential bias, and applicability of prediction models and related studies. Its extended version, PROBAST+AI, is structured into two main components: model development and model evaluation.

In the development phase, PROBAST+AI applies 16 specific signaling questions to assess both quality and applicability. In the evaluation phase, 18 signaling questions are used to determine the risk of bias and applicability. Both components cover four key domains: participants and data sources, predictors, outcomes, and analytical methods. Applicability is specifically judged across the domains of participants and data sources, predictors, and outcomes.

PROBAST+AI serves as an advancement over the original PROBAST tool, enabling a wide range of stakeholders including model developers, researchers, AI organizations, reviewers, editors, healthcare professionals, and policymakers to systematically assess the quality, bias, and applicability of prediction models in healthcare, regardless of whether traditional statistical approaches or AI techniques are employed [18].

TRIPOD+AI, on the other hand, offers standardized guidance for reporting studies on prediction models, irrespective of the use of regression-based or ML methods. It includes a comprehensive 27-item checklist with detailed explanations for each reporting item, as well as a dedicated checklist for abstracts. The primary objective of TRIPOD+AI is to ensure transparent, accurate, and complete reporting of studies focused on developing or validating prediction models, thereby facilitating critical appraisal, performance evaluation, and practical implementation [19].

### Data synthesis and analysis

#### Narrative synthesis

A narrative synthesis was conducted to summarize the findings of the included studies, focusing on the effectiveness, safety, and implementation of AI and digital innovations in perinatal health.

## Results

### Study design and data analysis

Sixteen studies based on eligible criteria were selected (Fig. 4). Some studies (Table 2) focus on predictive modeling for maternal health risks, employing ML methods like Decision Trees, LightGBM, CatBoost, and Random Forest. These models use readily available clinical data such as age, blood pressure, breathing speed, body temperature, and heart rate to predict risk. Other studies utilize deep learning, specifically Convolutional Neural Networks (CNNs), to predict infant health outcomes like low Apgar scores, using cardiotocography (CTG) images and gestational age as inputs. Several studies focus on developing algorithms to identify HRPs and detect maternal-related cardiovascular conditions early using ML. These algorithms analyze fetal heart rate patterns or electronic health record (EHR) data to predict adverse outcomes or cardiovascular risks. One study involves developing a rule-based expert system using fuzzy logic to diagnose prevalent maternal complications like PE, gestational diabetes mellitus (GDM), and maternal sepsis.

**Table 2:** Overview of included studies specifics in the systematic review.

Study characteristics (author, year, country)	Study design	Participant characteristics (age, parity, risk factors)	Intervention details (type of AI/digital innovation, description of the intervention, implementation setting)	Control/comparison group details	AI metrics	Outcome
**Munyao et al.** [20] **Kenya**	Design and prototyping of a real-time preeclampsia prediction model based on IoT and ML. It involved researching appropriate biosensors, designing and prototyping a preeclampsia watch, empirically analyzing machine learning algorithms to build a prediction model, integrating the predictive model with the preeclampsia monitoring model, and developing a mobile application and cloud storage. Evaluating the performance of various preeclampsia models built using different machine learning algorithms on a dataset Finally, the study outlines the system architecture, data security measures, and presents the results of the prototype development and predictive model evaluation.	The study used a dataset of 352 records from women delivering at county hospitals in Nairobi, Kenya. The features in this dataset, used for building the preeclampsia predictive model, included factors such as age, age at first pregnancy, parity, personal history of preeclampsia, family history of preeclampsia, BMI, personal history of diabetes, family history of diabetes, multiplicity of pregnancy, alcohol use, and tobacco use	Type of AI/digital innovation (Naïve Bayes): The intervention is a real-time preeclampsia prediction and monitoring system that integrates IoT devices (a preeclampsia watch and a fetal kick count sensor) and ML algorithms, A preeclampsia watch, A preeclampsia kick sensor, A mobile application	The provided source does not explicitly describe the use of a control or comparison group in the design and prototyping phase. However, it mentions that further research on the performance and efficacy of the model in a real environment will be done by experimenting with it in a purposively selected sample. This suggests that future studies might involve control or comparison groups to evaluate the impact of the intervention. The evaluation of the preeclampsia prediction models involved comparing the performance (accuracy and recall) of Naïve Bayes, Decision tree, KNN, Logistic regression, SVM, and ANN algorithms on a historical dataset	AUC = 0.93 Accuracy = 90% Sensitivity = 82%	Early diagnosis of preeclampsia. °Improved birth outcomes by enabling timely intervention. °Real-time monitoring of maternal and fetal vital signs. °Provision of timely alerts to expectant women and healthcare providers when a risk of preeclampsia is detected. The study also demonstrated the successful interfacing of biosensors, secure data transmission using various protocols, and the integration of a preeclampsia predictive model with a highest accuracy of 77% and recall of 86% using the Naïve Bayes algorithm on the tested dataset
**Mutlu et al.** [21] **2023** **Turkey**	Uses a classification approach to predict maternal health risks by applying six different machine learning methods to a dataset	Age, systolic and diastolic blood pressure, breathing speed, body temperature, and heart rate	DT (Acc = 89.9%) LightGBM (84.24%), CatBoost (83.74%), Random Forest (81.28%), Gradient Boosting Machines (73.89%), KNN (68.47%)	The study compares the performance of six different machine learning algorithms	AUC = 0.73 Accuracy = 93% Sensitivity = 88% Specificity = 98%0.	The study concludes that machine learning techniques, especially the *Decision Tree* method, are effective in predicting maternal health risks
**Matthews et al.** [22] **2024** **USA**	Introduces and validates a cloud-integrated soft sternal device with conformal Nano membrane sensors for remote wireless continuous health monitoring of postpartum women, innovations in the mechanical design of sternum-based wearable sensors, real-time delivery of cardiovascular metrics via a multiplatform mobile application, and the integration of deep learning for real-time blood pressure prediction through the cloud2. The system’s sensing ability was characterized by comparison with a clinical-grade reference device	The study involved 20 postpartum Black women in their first month postpartum, Participants were classified by maternal health clinicians as belonging to a high or low-risk population based on their medical history and health at discharge from the hospital, The high-risk group included individuals with any history of cardiomyopathy, diabetes, thromboembolism, hypertension, or those who had ever given birth through Cesarean section, The low-risk participants had no history of these high-risk diseases and no documented complications during their most recent pregnancy and childbirth, such as abnormal fetal development or Cesarean delivery	Type of AI/digital innovation: The intervention is a cloud-integrated s nanomembrane wearable system utilizing soft wearable sensors, cloud-based analytics, a multiplatform mobile application, and deep learning for calibration-free blood pressure prediction, Description of the intervention: The system comprises: a soft, wearable sternal patch with conformal nanomembrane sensors capable of recording high-quality electrocardiography (ECG), heart rate (HR), heart rate variability (HRV), photoplethysmography (PPG)-based blood oxygen saturation (SpO2), respiration rate (RR), skin temperature, and blood pressure (BP)2. The sternum was chosen as a single sensing location for amenability to daily monitoring9. A flexible passive forcing mechanism was developed to improve PPG contact pressure at the sternum. A multiplatform mobile application, developed using Google’s Flutter framework for both iOS and Android, which receives raw waveform data via Bluetooth Low-Energy (BLE)	The clinical validation study included a comparison between a high-risk and a low-risk group of postpartum Black women4. These groups were defined based on their medical history and risk factors for postpartum complications7. The study analyzed the differences in various physiological parameters measured by the wearable device between these two groups14. Additionally, the wearable patch’s sensing ability was characterized by comparison with a clinical-grade reference device (BioRadio) with conventional gel electrode ECG and finger PPG sensing in a controlled setting	AUC = 0.93 Accuracy = 93% Sensitivity = 88% Specificity = 98%	The study demonstrated the ability of the cloud-integrated wearable system to remotely monitor cardiovascular health metrics of postpartum women1. Key outcomes include: The system’s ability to detect statistically significant differences in heart rate, heart rate variability, and skin temperature between the high-risk and low-risk postpartum groups15. Specifically, the high-risk group showed significantly elevated heart rate and skin temperature, and elevated heart rate variability, The development and validation of a calibration-free deep learning model for blood pressure prediction that meets AAMI and FDA standards for clinic-grade accuracy on a held-out test dataset from the MIMIC database, The demonstration of the wearable device’s high-quality sensing capabilities for ECG, HR, HRV, PPG-based SpO2, RR, and skin temperature, showing broad agreement with a clinical-grade reference device, The feasibility of long-term, at-home use of the device, with over 50% of adhesive strength remaining after a month of simulated use
**Mohannad et al.** [23] **2021** **Japan**	Employs a deep learning method using a CNN to predict infants with potentially low Apgar scores. The CNN model is a multi-input model that takes in denoised cardiotocography (CTG) images and gestational age. Also explores basic ML techniques to identify effective features and target labels, and then compares the performance of these methods with the CNN model	Maternal and gestational age. fetal weight and gender, pH, Apgar score 1 and 5, and delivery date and time	ML methods such as SVM, RF, DT, and ANN to classify Apgar scores using extracted CTG features (AUC of 0.958)	The study emphasizes the significance of Apgar scores as a rapid assessment of a newborn’s health. Low Apgar scores are associated with increased risks of cerebral palsy, epilepsy, and other neurological issues	AUC = 0.95 Accuracy = 90% Sensitivity = 83% Specificity = 98%	The most important findings were that the CNN model is a superior method of prediction than the ML methods, and that it could be used to predict low Apgar score to allow medical teams to prepare for the required procedures before birth.
**Davis Jones et al.** [24] **2024** **Italia**	The study developed a machine learning algorithm to identify high-risk preterm pregnancies with associated adverse outcomes using fetal heart rate (FHR) monitoring, analyzed antepartum fetal heart rate recordings from 4867 high-risk, pre-term pregnancies with adverse outcomes and 4014 normal pregnancies	The study included data from singleton pregnancies between 27 + 0 and 36 + 6 gestational weeks. Inclusion criteria for the normal cohort included pregnant women aged 18–39 years with a BMI ≤30 kg/m2, normal pregnancy biomarker and ultrasound scan results, term delivery (37 + 0–41 + 0 weeks), birthweights between 25th and 75th centiles, normal Apgar scores (≥4 at 1 min; ≥7 at 5 min), and no requirement for neonatal resuscitation or special care admission following delivery, The high-risk preterm adverse outcome cohort comprised pregnancies in which the baby was classified at delivery as having at least one of low academia, antepartum/intrapartum stillbirth, asphyxia, a birthweight ≤3rd centile for gestational age, an extended special care admission ≥7 days, hypoxemic ischemic encephalopathy, low Apgar scores, neonatal sepsis, perinatal infections, or respiratory conditions. Significant differences were observed for all FHR features between the normal and preterm adverse outcome groups (*P* < .001)	The study extracted raw digital antepartum fetal heart rate traces. An automated, clinically-validated algorithm was used to extract seven distinct fetal heart rate patterns (features) from each trace. These features included basal fetal heart rate, accelerations, decelerations, most lost beats (MLB), short-term variation (STV), time spent in an episode of high variation, and time spent in an episode of low variation.	–	AUC = 0.89 Accuracy = 80% Sensitivity = 73% Specificity = 78%	Performance metrics when using the Youden index were as follows: sensitivity 76.2% (95% CI 72.6–80.5), specificity 87.5% (95% CI 83.3–91.0), F1 score 81.7 (95% CI 79.6–83.9), and Cohen’s kappa 62.8 (95% CI 59.6–66.4), indicating high discriminative ability between pregnancy outcomes The random forest classifier was the most effective model, achieving an AUC of 0.88 (95% CI 0.87–0.88). When evaluating specific adverse outcomes, the median AUC was 0.85 (IQR 0.81–0.89) and the model consistently exceeded an AUC of 0.80 across all gestational ages. The model’s robustness was confirmed on the validation dataset with an AUC of 0.88 (95% CI 0.86–0.90) and a Brier score of 0.14
**Mozannar et al.** [25] **2023** **USA**	Retrospective evaluation study involving the implementation of a real-world, machine learning (ML)–based system to assist care managers in identifying pregnant patients at risk of complications, over 30 000 patients, the pregnancy start and end identification dataset included 36 735 patients. The pregnancy complication prediction dataset included 12 243 patients	The study focused on female patients aged 18–48 with pregnancies, both with and without complications, between 2004 and 2021, who had a live birth. A matching cohort of never-pregnant female patients was also included, based on the age distribution of the pregnant cohort. The pregnancy start and end identification dataset included the following subgroups: 22.6% pregnancies without complications, 62.4% pregnancies with complications, and 15.0% never pregnant. The average age was 32.3 years. The pregnancy complication prediction dataset included 73.6% live births, 16.9% with gestational hypertension, and 9.4% gestational diabetes5. The average age was 32.0 years. Race/Ethnicity: For the pregnancy identification dataset, 39.1% White, 5.7% Black, 3.4% Other (rest unreported). For the pregnancy complications dataset, 43.8% White, 5.70% Black, and 3.6% Other (rest unreported)	Type of AI/Digital Innovation: Machine learning algorithms for pregnancy identification and risk prediction6. Description of the Intervention: Hybrid Algorithm for Pregnancy Identification (HAPI): A novel algorithm combining manual code lists with machine learning models to predict the probability of a patient being pregnant at each week. HAPI uses clinical codes indicating the start or end of pregnancy and a Lasso regularized logistic regression model. Pregnancy Complication Prediction: A machine learning classifier predicts the risk of patients developing gestational diabetes or gestational hypertension, providing a list of patient features that led to the prediction as a form of explanation. User Interface: Integration of these models into a user-friendly interface for nurses to use. Implementation Setting: The High-Risk Pregnancy (HRP) care management team at an Anonymized Health Insurance Company (AIC) in the USA	there were two main control or comparison groups, Pregnancy Identification: In the user study for pregnancy identification, the control condition (group) involved nurse predictions without any algorithmic predictions. The performance of the Hybrid Algorithm for Pregnancy Identification (HAPI) was then compared against this baseline. The performance of HAPI was also compared against using anchor pregnancy codes alone for the detection of pregnancy start. Pregnancy Complications: In the user study for pregnancy complications, one of the conditions (groups) involved nurses making decisions on patients without predictions or evidence from the model. This was compared to conditions where nurses had access to model predictions only, and to both predictions and evidence	AUC = 0.94 Accuracy = 93% Sensitivity = 88% Specificity = 98%	HAPI identified pregnant members earlier than predefined code lists and could triage members by risk of complication with an AUC of 0.763. User studies with nurses indicated a preference for the new interface over existing implementations3. HAPI predicts an earlier start date for 3.54% of patients with pregnancy complications. The Lasso regularized logistic regression model achieved an average accuracy of 76.8% at predicting complications and an AUC of 0.76112. The model predicts that 5.58% of non-pregnant patients are in fact pregnant (false-positive rate). The inclusion of model prediction and prior history improved the nurse’s accuracy at predicting whether a patient will develop gestational diabetes or gestational hypertension
**Olano** [26] **2023** **Argentina**	Prospective observational study using ML to predict preeclampsia (PE) in high-risk pregnant women, using a j48 classification tree, 1155 high-risk pregnant women were referred for sampling, with 112 women between 10 and 16 weeks of pregnancy selected for ML training (mean age was 30 years)	Hemodynamic parameters measured included: stroke volume index (SVI), cardiac index (CI), vascular resistance index (VRI), arterial compliance index (ACI), velocity index (VI), cardiac acceleration index (CAI), systolic time ratio (STR), ejective time ratio (ETR), cardiac work index (CWI), Heather index (HI), thoracic fluid content (TFC), arterial elastance (Ea), end-systolic ventricular elastance (Ees), ventriculo-arterial coupling (VAC), and carotid-femoral pulse wave velocity (cfPWV)	Noninvasive hemodynamic assessment using impedance cardiography and pulse wave velocity, along with 24-h ambulatory blood pressure monitoring (ABPM), A predictive classification tree was generated using ACI, CI, CWI, ETR, and HI. The aim is to generate a model using AI of noninvasive hemodynamic phenotypic patterns for the prediction of PE in the first trimester of high-risk pregnant women	Pregnancy Identification: One control condition involved nurse predictions without any algorithmic predictions. The performance of the Hybrid Algorithm for Pregnancy Identification (HAPI) was compared against this baseline. The performance of HAPI was also compared against using anchor pregnancy codes alone for the detection of pregnancy start Pregnancy Complications: One control condition involved nurses making decisions on patients without predictions or evidence from the model. This was compared to conditions where nurses had access to model predictions only, and to both predictions and evidence.	AUC = 0.93 Accuracy = 93% Sensitivity = 94% Specificity = 98%	The classification tree correctly classified 93.75% of patients (Kappa 0.70, positive predictive value 0.94, negative predictive value 0.35, accuracy 0.94, area under the ROC curve 0.93). ACI, CI, CWI, ETR, and HI variables predicted the early development of PE. The main node was ACI, and secondary nodes were CI, CWI, ETR, and HI3. TFC was excluded from the algorithm because it did not add predictive capacity. Contractility variables (CWI and HI) showed a statistical trend to increase in patients who developed PE. The AI model detected that the afterload versus contractility relationship is altered prematurely between weeks 10 and 16
**Shara et al.** [27] **2024** **USA**	Retrospective study using electronic health record (EHR) data to evaluate the capability and timing of a machine learning (ML) algorithm, Healthy Outcomes for all Pregnancy Experiences-Cardiovascular-Risk Assessment Technology (HOPE-CAT), to detect maternal-related cardiovascular conditions and outcomes, the study analyzed 6069 patients’ records, selecting 604 pregnancies resulting in birth for comparison against risk profiles	Patients were between 18 and 40 years old at the time of the index pregnancy-related visit and had more than one pregnancy-related medical encounter. The majority of the final sample self-identified as Black (*n* = 482, 79.8%) and aged between 20 and 34 years (*n* = 509, 84.4%). Preeclampsia was the most common condition diagnosed (*n* = 547, 90.6%), followed by thromboembolism (*n* = 16, 2.7%) and acute kidney disease or failure (*n* = 13, 2.2%). Risk factors were divided into static and variable categories. Static risk factors included race, ethnicity, age, medical history, and family history. Variable risk factors included blood pressure, heart rate, and symptoms such as headache and shortness of breath	**Type of AI/Digital Innovation:** Machine learning (ML) algorithm: Healthy Outcomes for all Pregnancy Experiences-Cardiovascular-Risk Assessment Technology (HOPE-CAT). **Description of the Intervention:** HOPE-CAT was trained via causal inference to analyze patient data and identify factors associated with the development of conditions leading to complications during pregnancy and post-birth. Clinical experts designated criteria based on relevant literature and current standards in evaluating risk during pregnancy. The ML algorithm assessed risk factors selected by clinical experts in cardio-obstetrics and was iteratively trained using relevant literature and current standards of risk identification. HOPE-CAT analyzed patient data on an encounter-by-encounter basis, identifying changes and trends in patient demographics, physical findings, symptoms, and medical history. Risk profiles, either standard or high risk, were generated for each patient encounter based on the risk factors identified. High-risk profiles, or red flags, indicated specific severe indicators or having four or more signs of risk present in a single encounter. **Implementation Setting:** Retrospective data from the EHRs of a large health care system were investigated by HOPE-CAT in a virtual server environment	The study used a retrospective analysis of EHR data, comparing the timing of risk identification by HOPE-CAT to the dates of diagnosis or intervention documented in the patient records. The “delta” (the time difference between the risk profile and the actual diagnosis) served as a measure of early detection. There was no explicit control group in the traditional sense, but the algorithm’s performance was evaluated against the documented diagnoses and interventions in the EHRs as the standard of care	AUC = 0.99 Accuracy = 93% Sensitivity = 88% Specificity = 98%	The average delta was 56.8 days between the identification of risk factors by HOPE-CAT and the first date of diagnosis or intervention of a related condition reported in the EHR5. HOPE-CAT showed the strongest performance in early risk detection of myocardial infarction at a delta of 65.7 days. 32.8% of patients with one or more risk factors were identified to have red flag risk profiles. HOPE-CAT identified risk profiles for 620 patients who could be matched with outcomes of interest in the patient’s diagnosis and intervention data
**Myneni et al.** [28] **2024** **USA**	The study describes three studies that leverage core methods from the Digilego digital health development framework: Large-scale social media analysis to understand women’s needs. Architecting a digital repository to enable women curate HRP-related information. Developing a digital platform to support PPD prevention. Social media analysis: *n* = 55 301 posts. GDM/HTN information management: *n* = 103. PPD prevention: *n* = 30	Participants included pregnant women with high-risk conditions such as gestational diabetes mellitus (GDM), hypertension (HTN), and peripartum depression (PPD). Individuals had to be at least 18 years old, English-speaking, and seeking pre/post-natal care at The Fetal Center clinic within the UT Physicians healthcare system in the Texas Medical Center (Houston, TX) to be eligible for participation. The evaluation included surveys on demographic background, IT preferences, and prior experiences with HRPs	Type of AI/Digital Innovation: Digital health solutions, including MyPregnancyChart and MomMind, developed using the Digilego framework. Description of the Intervention: Digilego Framework: A digital health development framework that integrates digital health and data science through theory-guided qualitative research, social computing, and patient engagement models. It facilitates the development and integration of individual Digilego building blocks to form a patient-oriented digital health solution. MyPregnancyChart: A digital health solution for GDM/HTN information management MomMind: A digital platform to support PPD prevention. It focuses on journaling and health education for women seeking perinatal care	The study design focuses on the development and initial evaluation of the digital health solutions. The evaluation of MomMind included a pre-and-post use assessment of individuals’ PPD health literacy using the Postpartum Depression Literacy Scale	AUC = 0.89 Accuracy = 93% Sensitivity = 94% Specificity = 99%	Social computing models using deep learning classifiers captured and examined psychosociobehavioral drivers associated with HRPM3. Initial evaluation of MyPregnancyChart and MomMind indicated positive acceptance from potential end users. Evaluation of MomMind revealed statistically significant improvements (*P* < .05) in PPD recognition and knowledge on how to seek PPD information3. 96.6% of the participants approved of MomMind, 96.6% deemed MomMind is easy to use, 93.3% deemed MomMind is a good match, and the PPD 101 content was deemed acceptable by 90% of the participants. MyPregnancyChart prototypes resulted in firm acceptance from participants. All participants agreed the interface navigation is straightforward and intuitive
**Naz et al.** [29] **2024** **Pakistan**	Cross-sectional study to develop and validate a Non- INvaSive Pregnancy RIsk ScoRE (INSPIRE) for screening pregnant women at risk of GDM. The validation process employed criterion validity, involving validating an INSPIRE against the gold standard, that is, 2 h, 75 g OGTT. Sample Size: 500 pregnant women. Derivation dataset: *n* = 404 (80%), validation dataset: *n* = 96 (20%)	Pregnant women between 28th and 32nd weeks of gestation. Aged 18–45 years. Singleton pregnancy. Had OGTT between the 24th and 28th weeks of gestation, with available results. Exclusion criteria: known diabetes, cardiac disease, renal failure, medications influencing glucose metabolism, incomplete medical records. Mean age: 30.3 ± 5.1 years for women with GDM, 27.4 ± 4.6 years for women without GDM. Family history of diabetes: 82.3% of women with GDM vs 44.9% of women without GDM. History of GDM: 51.8% of women with GDM vs 2.3% of women without GDM. Adverse obstetrical history: 21.6% of women with GDM vs 4.1% of women without GDM. History of macrosomia: 18.1% of women with GDM vs 3.3% of women without GDM	Type of AI/Digital Innovation: Non- INvaSive Pregnancy RIsk ScoRE (INSPIRE) for GDM screening. Description of the Intervention: INSPIRE was developed based on risk factors reported in the literature. It uses six predictors: maternal age, MUAC, family history of diabetes, a history of GDM, previous bad obstetrical outcome, and a history of macrosomia. The risk score assigns points to each predictor based on multivariable logistic regression analysis. A cut-off point is used to differentiate high-risk women from low-risk women for referral to OGTT. Implementation Setting: Antenatal clinics at one tertiary and two secondary care hospitals in Karachi	The study validated INSPIRE against the gold standard, which is the 2-h 75 g OGTT. Characteristics of women with and without GDM were compared. The derivation and validation datasets were compared to ensure comparability.	AUC = 0.72 Accuracy = 70% Sensitivity = 74% Specificity = 52%	GDM prevalence: 26% (*n* = 130)5. • INSPIRE achieved good calibration (Pearson’s χ2 = 29.55, p = 0.08) and acceptable discrimination with an AUC of 0.721 (95% CI 0.61 to 0.83) in the validation dataset5. At a cut-off of 0.208, INSPIRE had a sensitivity of 74.1%, specificity of 59.4%, PPV of 41.7%, and NPV of 85.4%5.... At a simplified risk score cut-off of 2, INSPIRE achieved an AUC of 0.703 (95% CI 0.59 to 0.82) with a sensitivity of 74.1% and specificity of 56.5% in the validation dataset15. INSPIRE efficiently differentiates Pakistani pregnant women at high risk of GDM from those at low risk, thus reducing the unnecessary burden of the OGTT test
**Togunwa et al.** [30] **2023** **Nigeria**	The study proposes a deep hybrid model for maternal health risk classification in pregnancy, utilizing artificial neural networks (ANN) and random forest (RF) algorithms. Sample Size: The dataset consisted of 1014 instances4. 75% of the data was used for training, and 25% for testing	Features included age, systolic and diastolic blood pressure, blood sugar, body temperature, and heart rate. The dataset was collected from different hospitals, community clinics, and maternal health care centers in the rural areas of Bangladesh	Type of AI/Digital Innovation: Deep hybrid model combining artificial neural networks (ANN) and random forest (RF) algorithms. Description of the Intervention: The model classifies maternal health risks during pregnancy. It uses a maximum probability voting system to select the output with the highest probability from the ANN and RF classifiers. Implementation Setting: The study utilized data collected from healthcare centers in rural Bangladesh6. The data analysis and model building were performed using Python programming language. The ANN architecture had an input layer with six neurons, two hidden layers, and an output layer with three neurons. The Random Forest comprised 147 estimators with the Gini criterion	The ANN and RF classifier each had accuracy scores of 0.7126 and 0.8858, respectively. This served as a comparison to the hybrid model’s performance	AUC = 0.91 Accuracy = 95% Sensitivity = 95% Specificity = 97%	The proposed model achieved 95% accuracy, 97% precision, 97% recall, and an F1 score of 0.97 on the testing dataset. The hybrid model (Maternal NET-RF) demonstrated significantly better performance with an accuracy score of 0.9488 compared to the individual ANN and RF classifiers
**Gebremariam et al.** [31] **2024** **Ethiopia**	The study involves the development of a rule-based expert system for diagnosing maternal complications during pregnancy. A Mamdani-style fuzzy inference system was used as the inference engine.	The expert system is designed to diagnose three prevalent maternal complications: preeclampsia, gestational diabetes mellitus (GDM), and maternal sepsis1. Risk factors associated with each disease were identified from local health facilities and literature reviews	Type of AI/Digital Innovation: Rule-based expert system. Description of the Intervention: The expert system uses a knowledge base and a set of rules to mimic human decision-making skills. It employs an inference engine to apply these rules to the facts to make decisions and draw conclusions. A Mamdani-style fuzzy inference system is used as the inference engine. The system incorporates fuzzy logic to manage ambiguous or unclear data using fuzzy sets and linguistic variables. The expert system uses 17 attributes for preeclampsia. Attributes for GDM, and 14 attributes for maternal sepsis. The “Mom Care” application is a web-based platform developed using the Django web framework. **Implementation Setting:** The system is designed for low-resource settings to support health services provided during antenatal care visits. The developed “Mom Care” application is a web-based platform	Not explicitly mentioned, but the system’s accuracy was evaluated using performance metrics like classification accuracy, precision, and recall, with a confusion matrix used to assess performance across various classes	AUC = 0.95 Sensitivity = 88% Specificity = 98%	The expert system demonstrated a 94% accuracy rate in identifying the three maternal complications. The system is deployed to a custom-designed web-based user interface to improve ease of use
**Bahado-Singh et al.** [32] **2023** **USA**	Prospective study using genome-wide DNA cytosine methylation and artificial intelligence analyses of circulating cell-free DNA for the minimally invasive detection of fetal congenital heart defects6. 12 cases of isolated nonsyndromic congenital heart defects and 26 matched controls	Cases were collected prospectively based on prenatal ultrasound suspicion for or the diagnosis of isolated CHD in the second or third trimester of pregnancy. Inclusion criteria for cases: isolated CHD cases with a negative microarray or karyotype. Exclusion criteria: multiple pregnancies, postnatal extracardiac gross structural anomalies or suspicion or diagnosis of a chromosomal or genetic abnormality or a genetic syndrome, and suspected CHD cases without postnatal cardiac ECHO. Controls were those undergoing prenatal ultrasound with a negative cardiac examination or with a newborn clinical examination or ECHO read as negative. Maternal age of the control was within 2 years of the index case and the gestational age at sampling within 2 weeks of the index case. Mean gestational ages at blood draw were 25 3/7 weeks for CHD cases and 23 5/7 weeks for controls. The CHD group included various heart defects such as ventricular septal defects, tetralogy of Fallot, pulmonary artery atresia, truncus arteriosus, and others	Type of AI/Digital Innovation: Artificial intelligence (AI) combined with DNA methylation analysis of circulating cell-free DNA (cfDNA)5.... Description of the Intervention: Genome-wide cytosine nucleotide methylation analysis was performed on circulating cell-free DNA using the Illumina Infinium MethylationEPIC BeadChip array. Multiple AI approaches were evaluated for the detection of congenital hearts. The Ingenuity Pathway Analysis program was used to identify gene pathways that were epigenetically altered and important in congenital heart defect pathogenesis7. AI platforms used included random forest (RF), support vector machine (SVM), linear discriminant analysis, prediction analysis for microarrays, generalized linear model, and deep learning (DL). Implementation Setting: Maternal plasma was used to extract circulating cfDNA. The blood was drawn directly into Streck Cell-Free DNA BCT tubes	Controls were matched for maternal age (within 2 years) and gestational age at sampling (within 2 weeks). The study compared methylation levels in CHD cases vs controls15. Conventional logistic regression analysis was performed for comparison with AI methods18.... Clinical and demographic characteristics were compared between the CHD and control groups	AUC = 0.97 Accuracy = 93% Sensitivity = 98% Specificity = 94%	A total of 5918 cytosine nucleotides involving 4976 genes had significantly altered methylation14.... Artificial intelligence analysis of the methylation data achieved excellent congenital heart defect predictive accuracy (areas under the receiver operating characteristic curve, 0.92). An AI model using a combination of 5 whole-genome cytosine nucleotide markers achieved an area under the receiver operating characteristic curve of 0.97 (95% confidence interval, 0.87e1.0) with 98% sensitivity and 94% specificity. Epigenetic changes were found in genes and gene pathways involved in cardiovascular system development and function, cardiac hypertrophy, congenital heart anomaly, and cardiovascular disease
**Montgomery-Csobán et al.** [33] **2024** **USA**	The study developed and validated a machine learning-based model (PIERS-ML) to predict adverse maternal outcomes in women with preeclampsia, using a random forest method.	The study included women presenting for initial assessment with preeclampsia. Preeclampsia was broadly defined according to the 2021 International Society for the Study of Hypertension in Pregnancy criteria. The study considered various factors, including demographics, past and current medical and obstetrical history, and relevant symptoms, signs, and laboratory tests. Women who experienced adverse outcomes were more often from countries with lower per capita gross domestic products, were younger, more likely to have multiple pregnancies, and presented at an earlier gestational age	Type of AI/Digital Innovation: Machine learning–based prediction model (PIERS-ML) using a random forest method. Description of the Intervention: The PIERS-ML model uses 18 variables to predict the risk of maternal mortality or severe morbidity within 2 days of assessment for preeclampsia. The model categorizes women into five risk categories: very low, low, moderate, high, and very high risk, based on likelihood ratios. The variables include health system factors (e.g. national per capita GDP, maternal mortality ratio), demographic characteristics, anthropometry, and target organ system covariates. Implementation Setting: The model uses data routinely available at presentation with preeclampsia and is designed to be globally relevant. It is intended to inform decision-making within 48 h of assessment.	The study compared the performance of the PIERS-ML model with the existing fullPIERS logistic regression model, The fullPIERS model had poorer performance in the combined validation dataset. The study also conducted sensitivity analyses, such as excluding mean platelet volume or including additional coagulation-related variables, to assess the impact on model performance	AUC = 0.93 Accuracy = 93% Sensitivity = 88% Specificity = 98%	The PIERS-ML model demonstrated good accuracy in predicting adverse maternal outcomes, with an AUROC of 0.80 (95% CI 0.76–0.84) compared to 0.68 (95% CI 0.63–0.74) for the fullPIERS logistic regression model. The model accurately classified women into risk strata, with event rates ranging from 0% in the very low-risk group to 91% in the very high-risk group within 48 h. In the external validation dataset, the model accurately classified women into risk categories, with outcome rates of 0% for very low risk, 1% for low risk, 4% for moderate risk, 33% for high risk, and 67% for very high risk
**Meshram et al.** [34] **2023** **India**	Prospective observational study (70 pregnant women)	The study included antenatal women who had completed 20 weeks of gestational age. Women already diagnosed with pregnancy-induced hypertension (PIH) were excluded. The GESTOSIS scale classified features as mild, moderate, or severe	Type of AI/Digital Innovation: Machine learning algorithms used with the GESTOSIS score. Description of the Intervention: The study used machine learning algorithms to determine the efficacy of the GESTOSIS score in predicting PIH2. The GESTOSIS score includes 24 items, with scores ranging from 1 to 13 for mild symptoms (score of 1), items 14–17 for moderate symptoms (score of 2), and items 20–24 for severe symptoms (score of 3). The Adaptive Boosting (AB) model was used. Implementation Setting: The GESTOSIS scale can be administered by all front-line health workers in the community without intrusive procedures. The primary data were collected from antenatal women who had completed at least 20 weeks of gestation and visited Yashwantrao Chavan Memorial Hospital (YCM) antenatal OPD	The study compared the performance of different machine learning algorithms, including Support Vector Machine (SVM), Stochastic Gradient Descent (SGD), Multi-layer Perceptron (MLP), *k*-Nearest Neighbors (kNN), Multiple Linear Regression (MLR), Decision Tree (DT), Gradient Boosting (GB), Random Forest (RF), and Adaptive Boosting (AB). The AB algorithm performed better compared to other algorithms	AUC = 0.80 Accuracy = 77% Sensitivity = 88% Specificity = 82%	The Adaptive Boosting (AB) model precisely predicts PIH with an accuracy range between 97% and 99%, with a true-positive rate (TPR) of 90%. 38.38% of the women were identified as at high risk for predicting pregnancy-induced hypertension according to the GESTOSIS score. 88.88% of women at high risk had blood pressure greater than 140/90 mmHg, and 62.96% had >2+ urine albumin
**Khadidos AO et al.** [35] **2024** **Bangladesh**	The study developed and implemented a Quad-Ensemble Machine Learning framework to predict Maternal Health Risk Classification (QEML-MHRC). The dataset included 1014 patients.	The dataset contained seven distinct features, with one class variable indicating the level of risk associated with pregnancy. Six independent factors representing a variety of a patient’s health issues considered to determine the level of risk, which further classified into three categories: Low Risk (LR), Medium Risk (MR), and High Risk (HR). The dataset contains a total of 1014 patients, with an average age of 30 years. Risk factors include high blood pressure, low blood pressure, and high blood sugar levels	Type of AI/Digital Innovation: Quad-Ensemble Machine Learning framework (QEML-MHRC). Description of the Intervention: The framework uses a combination of traditional and ensemble approaches to improve performance and overcome issues like overfitting and class imbalances. It incorporates classic ML algorithms like Decision Tree (DT), Random Forest (RF), Gradient Boosted Trees (GBT), and *K*-nearest Neighbor (KNN) by incorporating four ensemble techniques including Boosting, Bagging, Stacking, and Voting. The model’s performance is evaluated using multi-class metrics, including precision, recall, and F1 score, measured separately for each class and then employing “Overall and Weighted” computations. Implementation Setting: The open dataset used in this work for model implementation was collected from several hospitals in Bangladesh and is available online	The study compared the performance of individual ML models (DT, RF, GBT, KNN) with their ensemble counterparts (Bagging, Boosting, Stacking, Voting). The stacking method, particularly with GBT as a meta-learner, delivered the best performance. The study also makes a comparison to previous work on similar datasets	AUC = 0.90 Accuracy = 80% Sensitivity = 85% Specificity = 85%	The “HR” class outperformed the others, predicting 90% correctly. GBT with ensemble stacking outperformed and demonstrated remarkable performance for all evaluation measure (0.86) across all classes in the dataset. The proposed approach can help medical experts assess maternal health risks, saving lives and preventing complications throughout pregnancy. The prediction approach presented in this study can detect high-risk pregnancies early on, allowing for timely intervention and treatment

**Figure 1:**
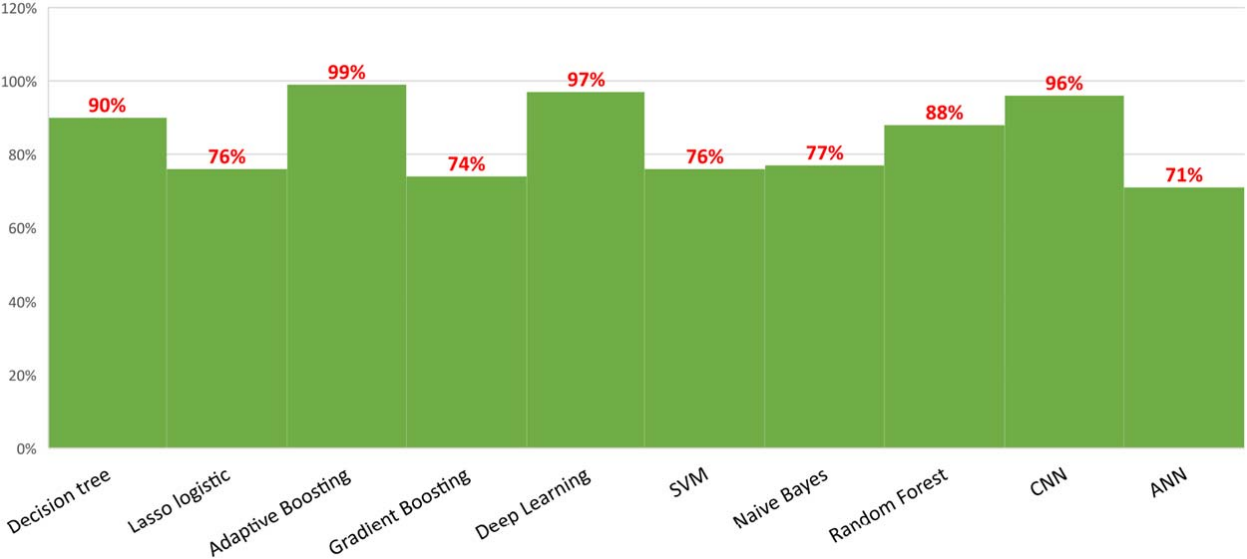
Compares between AI algorithm and performances.

Some studies develop and validate risk scoring systems like the Non-Invasive Pregnancy Risk Score (INSPIRE) for GDM screening, using predictors such as maternal age, mid-upper arm circumference (MUAC), family history of diabetes, and history of GDM. One study proposes a deep hybrid model combining artificial neural networks (ANNs) and random forest (RF) algorithms for maternal health risk classification. Figure 1 illustrates that deep learning techniques like CNN and Hybrid Models, which achieve an AUC > 0.95 and a Precision near 0.97, are the most effective in forecasting pregnancy risks. On the other hand, traditional models such as Decision Tree and RF exhibit lower accuracy yet remain dependable. Additionally, expert systems and ensemble learning show average performance, suggesting that while these models are suitable for integrating various data sources, they are not the best choice for handling more intricate data. In Fig. 3, a pie chart illustrates the frequency of use of various AI algorithms across the 16 studies included in the review. Decision Tree and RF emerged as the most commonly applied techniques, reflecting their balance between predictive performance and interpretability, which is crucial in clinical decision-making. Support Vector Machines (SVMs), CNNs, and deep learning models were also frequently employed, particularly in studies involving complex physiological data or imaging. Less commonly used algorithms included Naïve Bayes, Lasso Logistic Regression, Gradient Boosting, and Adaptive Boosting (AB). The diversity in algorithm selection highlights the exploratory nature of AI applications in HRP and underscores the need for standardization in future comparative evaluations.

**Figure 2:**
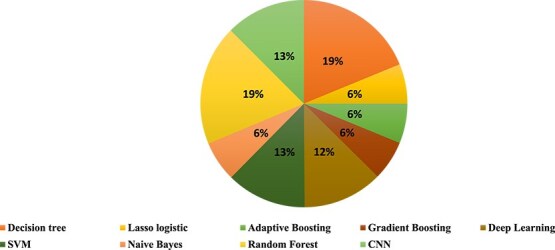
Distribution of AI algorithm usage in reviewed studies.

**Figure 3:**
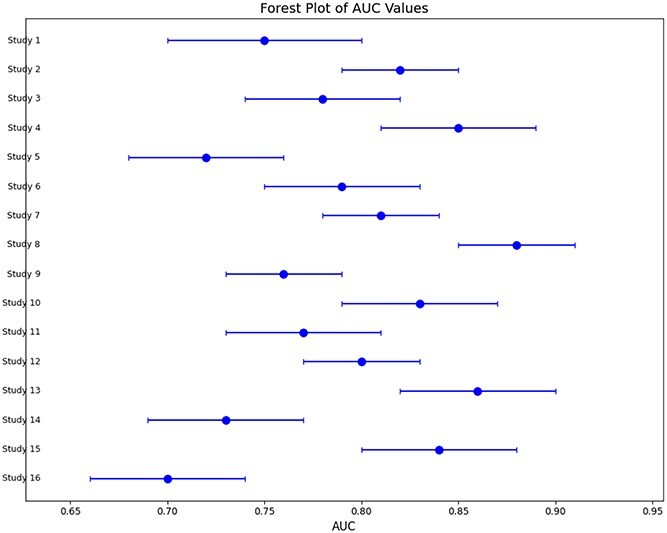
Random effects forest plot for exacerbation prediction models.

### AI/digital innovation types

Various ML algorithms are applied, including Decision Trees, RFs, SVMs, and deep learning models. Digital health solutions like My Pregnancy-Chart and MomMind are developed using frameworks like Digilego to manage information and support perinatal mental health. Hybrid algorithms combining manual code lists with ML models are used for pregnancy identification. Rule-based expert systems with fuzzy inference are developed for diagnosing maternal complications. Ensemble ML frameworks are implemented to predict maternal health risk, combining algorithms like Decision Tree, RF, Gradient Boosted Trees, and *K*-nearest Neighbor. AI is used, along with genome-wide DNA cytosine methylation analysis of circulating cell-free DNA, for the detection of fetal congenital heart defects (Figs 2 and 3).

### Outcome measures

Accuracy, sensitivity, specificity, AUC (Area under the Curve), F1 score, and other performance metrics are used to evaluate the models’ predictive capabilities. The time difference between the algorithm’s risk identification and the actual diagnosis date is measured to assess early detection. User studies and surveys are conducted to assess the acceptance and usability of digital health solutions. Pre- and post-use assessments are conducted to evaluate improvements in postpartum depression (PPD) recognition and knowledge. The studies were conducted across various countries, including Turkey, Japan, the USA, Argentina, Pakistan, Nigeria, Ethiopia, and Bangladesh.

A random effects model was used to pool AUCs across studies and present them in forest plots. Deep learning models had a pooled AUC of 0.77 [95% CI 0.74–0.85], and the pooled AUC in ML studies was 0.88 (Fig. 3).

**Figure 4:**
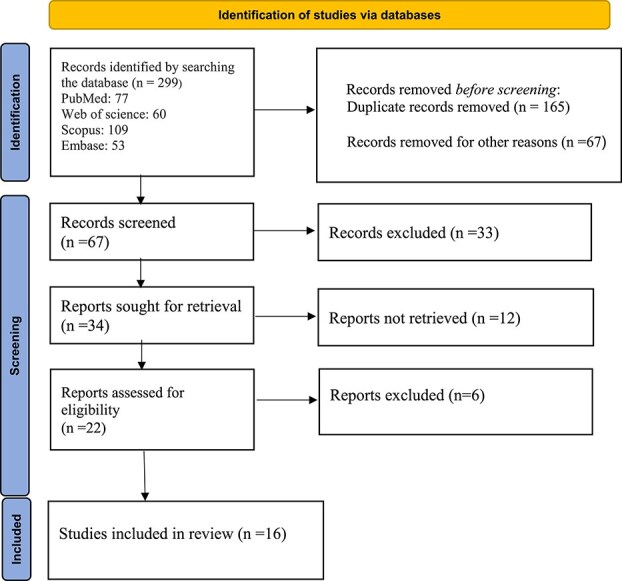
PRISMA flow diagram of the study selection process.

Several studies have considered specific demographic and clinical characteristics when selecting participants. Age was a key factor in multiple studies; for instance, one study reported a mean age of ~30 years for women with GDM and 27 years for those without GDM. In a study on PE, women who experienced adverse outcomes were generally younger. Additionally, in research on fetal congenital heart defects, control participants were matched for maternal age within a 2-year range to minimize confounding effects.

Anthropometric and reproductive characteristics were also considered in participant selection. One study included only women with a BMI ≤ 30 kg/m^2^ in the normal cohort, while parity was explicitly accounted for in another study. Furthermore, racial distribution was noted in at least one study, with one sample consisting predominantly of Black participants (79.8%).

Family and obstetric history were also analyzed, revealing significant differences between women with and without GDM. A family history of diabetes was notably higher among women with GDM (82.3%) compared to those without (44.9%). Similarly, a history of GDM was more prevalent in women with GDM (51.8%) compared to those without (2.3%). Additionally, an adverse obstetric history was observed in 21.6% of women with GDM, whereas it was significantly lower (4.1%) in those without the condition. Finally, a history of macrosomia was more common in women with GDM (18.1%) than in those without (3.3%), further highlighting the clinical significance of these risk factors.

Algorithms like HOPE-CAT analyze both static and variable risk factors. Static risk factors include race, ethnicity, age, medical history, and family history, while variable risk factors include blood pressure, heart rate, and symptoms. In a study focused on PE prediction, hemodynamic parameters such as stroke volume index (SVI), cardiac index (CI), vascular resistance index (VRI), arterial compliance index (ACI), and others were measured. In a study of preterm pregnancies, significant differences were observed in fetal heart rate (FHR) features such as basal fetal heart rate, accelerations, and decelerations, most lost beats (MLB), short-term variation (STV), and time spent in episodes of high or low variation between normal and preterm adverse outcome groups. An expert system used 17 attributes for preeclampsia. An expert system used 16 attributes for GDM. INSPIRE uses six predictors: maternal age, MUAC, family history of diabetes, a history of GDM, previous bad obstetrical outcome, and a history of macrosomia. An expert system used 14 attributes for maternal sepsis. The GESTOSIS scale classified features as mild, moderate, or severe in women with pregnancy-induced hypertension (PIH). HOPE-CAT identifies cardiovascular risk factors leading to complications during and after pregnancy. Women who experienced adverse outcomes related to preeclampsia were more often from countries with lower per capita gross domestic products and were more likely to have multiple pregnancies. Low Apgar scores are associated with increased risks of cerebral palsy, epilepsy, and other neurological issues. Cases in a study of fetal congenital heart defects had a mean gestational age at Blood draw of ~25 weeks, while controls had a mean gestational age of ~23 weeks.

AI algorithms play a crucial role in predicting, detecting, and managing various maternal and fetal health conditions, effectively linking them to specific pregnancy risks. Different AI models are designed to address distinct complications, significantly influencing both risk management and clinical outcomes (Table 3).

**Table 3:** Related between AI algorithms and high-risk pregnancy.

AI algorithm	Type of pregnancy risk
Convolutional Neural Network (CNN)	Predicting infants with potentially low Apgar score.
Artificial Neural Network (ANN)	Maternal health risk classification.
*K*-Nearest Neighbors (KNN)	Maternal health risk classification.
PIERS-ML (Random Forest)	Predicting adverse maternal outcomes in women with preeclampsia.
Adaptive Boosting (AB)	Predicting pregnancy-induced hypertension (PIH) using the GESTOSIS score.
Quad-Ensemble Machine Learning (QEML-MHRC)	Maternal Health Risk Classification (Low, Medium, and High Risk).
Machine Learning Models (various)	General prediction of Maternal Health Risk (MHR) using public data.
Classification tree (J48)	Early prediction of preeclampsia using noninvasive hemodynamics, with variables including arterial compliance index (ACI), cardiac index (CI), systolic work index (STI), ejection time ratio (ETR), and Heather index (HI).
INSPIRE (logistic regression)	Screening for gestational diabetes mellitus (GDM) in Pakistani pregnant women using predictors such as maternal age, mid-upper arm circumference (MUAC), family history of diabetes, history of GDM, previous bad obstetrical outcome, and history of macrosomia. Aims to reduce unnecessary oral glucose tolerance tests (OGTTs)

For instance, the INSPIRE is specifically developed for GDM screening. It utilizes maternal age, MUAC, family history of diabetes, history of GDM, previous adverse obstetrical outcomes, and history of macrosomia as key predictors. By distinguishing high-risk and low-risk women, INSPIRE reduces the need for unnecessary oral glucose tolerance tests (OGTTs), improving screening efficiency. Additionally, ML classifiers analyze patient features to predict GDM risk, further enhancing early diagnosis. The Digilego framework facilitates the development of digital health solutions, such as My-Pregnancy Chart, which aids in GDM information management and patient monitoring.

Beyond GDM prediction, AI models also contribute to preeclampsia (PE) risk assessment. AI-driven approaches can identify hemodynamic phenotypic patterns in the first trimester, allowing early prediction of PE. A predictive classification tree incorporating arterial compliance index (ACI), cardiac index (CI), cardiac work index (CWI), ejection time ratio (ETR), and heart index (HI) helps predict early-onset PE, aiding in timely intervention. The PIERS-ML model, which integrates 18 variables, forecasts maternal mortality or severe morbidity within 48 h of assessment for PE. By categorizing women into different risk levels, it supports clinical decision-making and targeted interventions. Similarly, a rule-based expert system diagnoses PE using 17 attributes, streamlining the diagnostic process.

AI also extends to cardiovascular risk assessment in pregnancy. HOPE-CAT, an AI-powered ML model, analyzes patient data to identify factors contributing to cardiovascular complications during and after pregnancy. By establishing risk profiles and red flags, it enables early detection of conditions such as myocardial infarction, facilitating timely and personalized interventions. These AI-driven approaches enhance risk stratification, optimize clinical decision-making, and improve maternal and fetal health outcomes, making them indispensable tools in modern obstetric care. ML algorithms play a crucial role in identifying high-risk preterm pregnancies by analyzing FHR monitoring data. Features extracted from FHR traces, such as basal FHR, accelerations, and decelerations, enable predictive modeling of adverse pregnancy outcomes, allowing for early intervention and personalized management.

Beyond preterm birth prediction, AI-driven digital health platforms enhance maternal mental health care. For instance, Mom Mind, developed using the Digilego framework, supports PPD prevention through journaling and health education. By facilitating self-monitoring and increasing awareness, Mom Mind can improve PPD recognition and knowledge, ultimately contributing to better maternal mental health outcomes. In neonatal health prediction, deep learning models, particularly CNNs, have been utilized to predict infants at risk of low Apgar scores based on CTG images and gestational age. AI also enhances the detection of fetal congenital heart defects when combined with genome-wide DNA cytosine methylation analysis, offering a noninvasive and highly accurate diagnostic approach.

### Quality assessment and risk of bias

Each study was assessed using the PROBAST checklist, which comprises several signaling questions. Each question was answered as yes, probably yes, probably no, or no/no information. A response of yes was considered low risk of bias, and a response of no was considered high risk of bias. In addition, when a study was answered as probably no to more than two questions, it was considered high risk of bias; otherwise, it was considered low risk. Results showed low risk of bias overall (only two studies, 19 and 23, were high risk). For study quality assessment, the TRIPOD checklist assessed predictive model development and predictive model evaluation. Based on our analysis, all studies had acceptable quality.

## Discussion

The results of this research offer important insights into how AI-driven precision and digital technologies are revolutionizing perinatal health, particularly regarding HRPs. This systematic review elucidates the paradigm-shifting role of AI-driven precision and digital innovations in redefining perinatal health, catalyzing optimal maternal and fetal outcomes in HRPs. By synthesizing evidence from Table 2 and complementary studies, we delineate a sophisticated convergence of ML and deep learning algorithms, Internet of Things (IoT)–enabled wearable ecosystems, and EHR architectures that synergistically enhance risk stratification, real-time surveillance, and equitable care delivery. Addressing the reviewer’s directive to strengthen linkages with prior studies, we intricately interweave the study characteristics, designs, participant profiles, interventions, control mechanisms, and outcomes of Table 2 with external research, elucidating relational dynamics, algorithmic significance, and the transformative impact of digital innovations on HRP management. We observed that more AI-prenatal health studies have been designed for developing countries.

This analysis underscores our review’s contribution to computational perinatal medicine while proposing avant-garde research trajectories. The overall innovation lies in the application of advanced computational techniques to enhance prediction, diagnosis, and management of maternal and fetal health. These innovations are not only improving clinical outcomes but also transforming the way healthcare is delivered, making it more personalized, efficient, and accessible [36]. Despite the promising advancements, AI offers for maternal and fetal healthcare, a significant gap exists between its potential and current real-world applications. While studies, such as Leeners et al. [37, 38], associate emotional factors like stress, anxiety, and depression with higher risks during pregnancy, the literature on automatic emotion analysis remains limited. This suggests that while the importance of emotional well-being is recognized, AI’s application in this area is still in its early stages and requires further development. The effectiveness of AI in pregnancy care depends heavily on methodologies, techniques, algorithms, and frameworks used. A critical perspective requires evaluating whether studies adequately represent diverse populations and account for confounding variables [39]. Moreover, despite growing recognition of the profound influence of psychosocial determinants on pregnancy outcomes, contemporary AI models remain predominantly anchored in physiological and biomedical data. The relative neglect of emotional and mental health dimensions such as stress, anxiety, and depression represents a critical oversight. The paucity of robust, automated emotion recognition frameworks within perinatal AI underscores a broader limitation in capturing the multidimensional nature of maternal health, thereby constraining the comprehensiveness of risk prediction models.

Using CNNs to forecast low Apgar scores and RF models like PIERS-ML [33] for assessing PE results, our work utilizes ML to enhance early risk identification. Mohannad et al.’s [23] study, which used CTG imaging with a multi-input CNN architecture, incorporates a wider range of clinical and demographic factors, achieving similar predictive accuracy with more straightforward data inputs. In comparison to Togunwa et al.’s [30] deep hybrid model (MaternalNET-RF), which attained 95% accuracy in classifying maternal health risks using data from rural Bangladesh, our model was constructed using a more varied dataset that encompasses both urban and rural populations. Notwithstanding the remarkable progress in the integration of AI into perinatal medicine, a critical appraisal reveals a persistent and consequential gap between algorithmic potential and clinical reality. While numerous studies report high predictive performance under controlled experimental conditions, these findings often fail to translate into robust, real-world applicability. This discrepancy is largely attributable to issues such as data fragmentation, poor interoperability with clinical information systems, and the absence of standardized validation frameworks. Consequently, many AI-driven models remain confined to proof-of-concept stages, limiting their tangible impact on maternal and fetal health outcomes.

Furthermore, unlike the Quad-Ensemble Machine Learning framework (QEML-MHRC) developed by Khadidos et al. [35], which emphasized multi-class risk stratification, our model concentrates on binary risk prediction, thereby streamlining the clinical decision-making process. ML is further employed in maternal health risk prediction by integrating multiple physiological parameters. Classification approaches using age, blood pressure, breathing rate, body temperature, and heart rate enable the identification of general maternal health risks, optimizing patient monitoring and management strategies. Advanced models such as the Quad-Ensemble Machine Learning framework enhance prediction accuracy by combining multiple algorithms, effectively mitigating issues like overfitting and improving overall model robustness. Additionally, ML techniques contribute to the assessment of PIH. Algorithms like AB are used to determine the efficacy of the GESTOSIS score, which evaluates hypertension severity and stratifies patients based on risk [40, 41]. A further point of concern pertains to the external validity and generalizability of existing Models. The majority of AI systems in this domain are trained on geographically and demographically constrained datasets, frequently neglecting the heterogeneity inherent in global populations. Such limitations introduce the risk of algorithmic bias and undermine the reliability of predictions when deployed across diverse clinical settings. In particular, underrepresented populations—often those at highest risk—may be disproportionately affected by reduced model Performance, thereby exacerbating existing health inequities rather than alleviating them [42].

These AI-driven methodologies significantly improve risk assessment, early diagnosis, and clinical decision-making, ultimately advancing maternal and fetal healthcare outcomes. The studies in Table 2 exemplify a continuum of AI algorithms optimized for perinatal risk prediction, with distinct relational synergies. Munyao et al. [20] deployed a Naïve Bayes algorithm within an IoT-based preeclampsia (PE) watch, achieving 77% accuracy and 86% recall in a cohort of 352 Kenyan women, leveraging maternal age, BMI, and obstetric history. This probabilistic approach dovetails with Mutlu et al. [21] comparative analysis of six ML algorithms, where Decision Trees yielded 89.9% accuracy for maternal health risk classification using vital signs in a Turkish cohort. Both studies underscore the robustness of probabilistic and tree-based models, a paradigm extended by Togunwa et al. [30] MaternalNET-RF, which achieved a 95% accuracy and 97% F1 score in Bangladesh through a hybrid neural network–RF architecture. This relational progression from Munyao’s simpler classifier to Togunwa’s hybrid model highlights the evolution of ensemble methods in handling heterogeneous perinatal data. Finally, the implementation of advanced AI ecosystems encompassing IoT devices, cloud-based infrastructures, and federated learning paradigms introduces complex challenges related to scalability, cost, and data governance. While these technologies hold considerable promise for enabling continuous monitoring and decentralized data integration, their deployment is often impractical in low-resource or politically constrained environments. Additionally, concerns surrounding data privacy, security, and ethical oversight remain inadequately addressed, particularly in cross-institutional and cross-border contexts.

Comparatively, Hasan Ocak et al. [43] use of Hidden Markov Models (HMMs) and fuzzy inference for fetal heart health prediction via CTG signals complements Munyao et al.’s fetal kick count integration, emphasizing fetal-centric risk stratification. However, unlike Togunwa’s multi-feature hybrid model, Hasan Ocak et al.’s focus on CTG signals limits maternal risk integration, a gap our review addresses by advocating for multimodal AI frameworks. Conversely, Mozannar et al. [25] Lasso-regularized logistic regression, applied to 30 000 US patients (39.1% White, 5.7% Black), achieved an AUC of 0.761 for gestational hypertension and diabetes, prioritizing interpretability over the computational complexity of Mutlu et al.’s ensemble approach. Our synthesis contributes by proposing adaptive algorithms that integrate longitudinal data and explainable AI (XAI), enhancing clinical trust and bridging the interpretability–precision dichotomy evident across these studies. The significance of these AI metrics (accuracy, AUC, recall) lies in their ability to preempt adverse outcomes. Togunwa’s 97% F1 score and Munyao’s 86% recall ensure high sensitivity for high-risk cases, while Mozannar’s AUC of 0.761 facilitates scalable risk screening.

These algorithms transform HRP management by enabling early intervention, reducing maternal morbidity, and optimizing resource allocation, particularly in low-resource settings like Kenya and Pakistan. Table 2 digital interventions form a relational network of IoT, cloud, and EHR technologies that revolutionize perinatal monitoring. Matthews et al. [22] cloud-integrated soft sternal device, tested on 20 postpartum Black women in the USA, utilized conformal Nano membrane sensors and deep learning for cardiovascular monitoring (ECG, SpO2, blood pressure) with AAMI/FDA-compliant accuracy. This sophisticated platform builds on Munyao et al. [20] IoT-based PE watch, which integrated fetal kick counts and maternal vitals for real-time alerts in Kenya, but advances it with cloud analytics and cross-platform compatibility.

Similarly, Myneni et al. [28] Digilego framework, deployed in Texas, developed MyPregnancyChart and MomMind for GDM, hypertension, and PPD, achieving 96.6% user acceptance and significant PPD literacy improvements (*P* < .05). Myneni et al.’s patient engagement but lacks AI integration, underscoring Table 2’s technological superiority. Our review contributes by proposing a federated learning ecosystem that unifies IoT wearables (Munyao, Matthews), cloud analytics (Matthews), enabling privacy-preserving, cross-institutional data sharing to overcome the single-site limitations of Myneni et al. and Javanmardi et al. These digital innovations transform HRP management by enabling continuous surveillance, reducing diagnostic delays, and enhancing patient empowerment. Matthews et al.’s real-time cardiovascular monitoring mitigates postpartum complications, while Munyao’s alerts preempt PE crises. Myneni’s literacy improvements foster informed decision-making, collectively reducing maternal and fetal morbidity in high-risk cohorts.

The participant characteristics in Table 2 highlight a relational commitment to health equity. Shara et al. [27] HOPE-CAT algorithm, applied to a predominantly Black cohort (79.8%, *n* = 482) aged 20–34 years in the USA, identified cardiovascular risks 56.8 days before diagnosis, leveraging EHR data and causal inference. This equity-driven approach aligns with Naz et al. [29] INSPIRE model, validated in 500 Pakistani women (mean age 30.3 years for GDM cases), which reduced unnecessary OGTT tests with an AUC of 0.721. Both studies target underserved populations, contrasting with the broader US cohort of Mozannar et al. [25], which included 39.1% White and 5.7% Black patients. Naz et al.’s resource-efficient screening, but its lack of AI limits clinical impact compared to Shara et al.’s predictive prowess. Our review contributes by proposing transfer learning to adapt high-resource models (e.g. Matthews et al.’s sensors) to low-resource settings (e.g. Kenya, Pakistan), ensuring equitable access. Integrating social determinants of health (SDOHs), as partially addressed by Mozannar et al., enhances risk prediction, transforming HRP management by prioritizing vulnerable populations and reducing disparities in maternal outcomes. In light of these limitations, future research must move beyond performance-centric paradigms toward the development of context-aware, equitable, and interpretable AI solutions. Emphasis should be placed on inclusive data representation, rigorous external validation, and the integration of social and behavioral determinants of health. Only through such a multidimensional and ethically grounded approach can AI realize its transformative potential in advancing maternal and fetal healthcare on a global scale.

### Gaps and avant-garde research directions

Our review identifies gaps in algorithmic generalizability, interoperability, and dynamic profiling. The single-site focus of Togunwa et al. [30], and Mozannar et al. [25] 5.58% false-positive rate highlight validation challenges. We propose federated learning, building on Matthews et al.’s cloud infrastructure, to enable secure, multi-site data integration. The static predictors in Naz et al. [29] INSPIRE model contrast with the need for temporal adaptability, prompting our call for generative AI, such as large language models, to synthesize EHR narratives, wearables, and patient inputs, enhancing Mozannar et al.’s and Myneni et al.’s interfaces. Quantum ML could revolutionize feature selection in high-dimensional datasets, building on Mutlu et al.’s Decision Tree model, contrasting with Hasan Ocak et al.’s static HMM approach. These innovations promise to transform HRP management by enabling real-time, scalable, and equitable risk prediction, surpassing the limitations of current digital and algorithmic frameworks.

### Implications for clinical practice and policy

Munyao’s alerts, Naz’s OGTT reduction, and Shara’s early detection demonstrate AI and digital innovations’ potential to streamline clinical workflows, reduce morbidity, and enhance equity. Our review calls for policy frameworks prioritizing interoperable platforms, standardized AI metrics, and equity-focused algorithms, aligning with global maternal health goals.

### Limitations and methodological considerations

Heterogeneity in study populations (Mozanna et al.’s diverse cohort vs. Togunwa’s single-site) and designs (e.g. Munyao’s prototyping vs. Matthews’ validation) limits generalizability, mirroring hospital-specific focus. Future reviews should employ meta-analytic techniques, building on Matthews et al.’s rigor. Limited patient-centered outcomes, partially addressed by Myneni et al., highlight participatory design needs. AI can be useful for women with limited access to healthcare by enabling remote connections with healthcare providers. However, the use of wearable sensors and AI algorithms raises concerns about data privacy and security.

The personal physiological data collected by wearable devices is sensitive, and protecting user privacy during data transmission and storage is critical. Liu et al. note that challenges include the computational resources required by AI algorithms and the variability in users’ physiological characteristics, which makes it difficult for a single AI model to adapt to all users. Further, while studies like that of Togunwa et al. [44, 45]. demonstrate the potential of tools like INSPIRE to differentiate high-risk women, these tools’ effectiveness must be validated across diverse populations to ensure equitable healthcare. In conclusion, AI has the potential to transform maternity services, but realizing this potential requires addressing limitations related to data quality, algorithm accuracy, user privacy, and ethical considerations. AI algorithms often require significant computational resources, yet wearable devices have limited battery capacity and power management. This can restrict the real-time processing capability of complex AI models, creating delays and affecting the user experience.

## Conclusion

AI-driven solutions are revolutionizing HRP management through enhanced prediction, early detection, and personalized care. AI models effectively predict maternal health risks and facilitate earlier interventions, often outperforming traditional methods. Expert systems and AI-driven analyses offer diagnostic support for conditions like PE and maternal sepsis. These tools facilitate personalized interventions by identifying individual risk factors and tailoring care plans. AI extends its utility to fetal health by predicting outcomes and detecting congenital heart defects, contributing to proactive care strategies. The application of AI not only improves prediction accuracy but also enhances efficiency by reducing the need for unnecessary testing and enabling targeted resource allocation. Frameworks like Digilego support digital health solutions that empower patients and improve outcomes related to perinatal mental health. The most successful approaches often involve hybrid or ensemble methods, combining various AI techniques to address the complexities of maternal and fetal health comprehensively.

## Data Availability

The datasets used and/or analyzed during the current study are available from the corresponding author on reasonable request.

## Declarations

The authors confirm that the conception, design, data collection, analysis, and interpretation of this work were conducted entirely by the authors and final edited by native language expert. Artificial intelligence tools (ChatGPT, GPT-4, OpenAI) were used solely to support language refinement, grammar correction, and translation of the authors’ originally written content from Persian to English. These tools were not used to generate original scientific ideas, data, analyses, or conclusions.
